# GABAergic System Dysfunction and Challenges in Schizophrenia Research

**DOI:** 10.3389/fcell.2021.663854

**Published:** 2021-05-14

**Authors:** Muhammad Jahangir, Jian-Song Zhou, Bing Lang, Xiao-Ping Wang

**Affiliations:** ^1^Department of Psychiatry, National Clinical Research Center for Mental Disorders, The Second Xiangya Hospital of Central South University, Changsha, China; ^2^School of Medicine, Medical Sciences and Nutrition, Institute of Medical Sciences, University of Aberdeen, Aberdeen, United Kingdom

**Keywords:** schizophrenia, GABAergic system, oscillations, stem cells, animal models

## Abstract

Despite strenuous studies since the last century, the precise cause and pathology of schizophrenia are still largely unclear and arguably controversial. Although many hypotheses have been proposed to explain the etiology of schizophrenia, the definitive genes or core pathological mechanism remains absent. Among these hypotheses, however, GABAergic dysfunction stands out as a common feature consistently reported in schizophrenia, albeit a satisfactory mechanism that could be exploited for therapeutic purpose has not been developed yet. This review is focusing on the progress made to date in the field in terms of understanding the mechanisms involving dysfunctional GABAergic system and loops identified in schizophrenia research.

## Introduction

Schizophrenia (SCZ) is a debilitating neurological disorder with a worldwide prevalence of 1% (Sullivan et al., 2003). It is only diagnosed on the basis of clinical symptoms including the following: (1) positive features: hallucinations (seeing, hearing, smelling, tasting, or feeling things that are actually not there), delusions (fixed false beliefs or suspicions that are firmly held even when there is evidence to the contrary), and thought disorders; (2) negative symptoms: social withdrawal, anhedonia, and blunted affect; and (3) cognitive deficit: executive function, attention, and working memory impairment (Nakazawa et al., 2012). Negative symptoms of SCZ appear several years before the first acute schizophrenic episode and are often referred to as the prodromal period of SCZ. Patients lack interest and motivation in life, withdraw socially, and do not care about their personal appearance. These symptoms evolve gradually and progressively worsen (Mueser and Jeste, 2008), but at this stage, usually it is difficult to differentiate from depression. Multiple genetic elements causing schizophrenia have been identified, but among them, the one gene completely responsible for the symptoms has not been found, which implicates that schizophrenia is consequenced by interactions of genetic, environmental, and developmental factors. Epigenetic alterations, genetic mutations, and cellular deficits disturb brain circuits. All these either individually or together result into clinical manifestations (Nakazawa et al., 2012). Therefore, much is still needed to investigate the mechanistic loop of schizophrenia pathophysiology. Understanding how multiple genes interact and precipitate a plethora of abnormal phenotypes and developing animal models that can mimic exactly the pathology can be helpful to unknot the complexities.

Unfortunately, the neuropathological finding of schizophrenia is subtle. A slight loss of brain volume has been so far consistently confirmed in growing literature. Interestingly, this reduction largely results from loss of neuropil rather than neuron numbers, potentially highlighting abnormal synaptic plasticity and cortical micro-circuitry. A meta-analysis of neuroimaging studies on patients with SCZ also confirmed the enlargement of lateral and third ventricles and smaller cortical and gray matter volumes (Nelson et al., 1998; Wright et al., 2000). Alterations in physiology, cytoarchitecture, brain structure, and molecular chemistry have all been reported for schizophrenia pathology, but none of them meets the criteria of a diagnostic marker for schizophrenia disease.

Though separate but linked, hypotheses for schizophrenia pathology include glutamatergic and dopaminergic hypotheses are being highlighted here. Clinical research suggest that glutamate level may alter prior to the onset of psychosis (Egerton et al., 2020), whereas glutamatergic elevation has also been evidenced in schizophrenia (Merritt et al., 2016). Presynaptic dopaminergic abnormality in schizophrenia affects dopamine synthesis capacity, dopamine release, and baseline synaptic dopamine level. While an elevation of postsynaptic D2 receptors has also been proposed, the findings of a meta-analysis have been less convincing (Howes et al., 2012). Furthermore, hypo-functioning of NMDA receptor causes disinhibition of glutamatergic projections, onto midbrain dopamine neurons, and increases glutamate release, which in turn enhances the activation of dopaminergic neurons. This implicates that dopamine dysregulation in schizophrenia could be secondary to glutamatergic dysfunction (Howes et al., 2015).

The hypo-functioning of NMDA type of glutamate receptor (NMDAR) has long been proposed as a key contributor to schizophrenia. It is believed that it may alter the excitability of GABAergic neurons and thus neural network oscillation, which promotes the occurrence of cognitive deficit. NMDAR hypofunction may directly reduce the tone of GABAergic interneurons, which leads to overexcitability of glutamatergic pyramidal neurons. The latter may result in an excessive stimulation by glutamate and promote neuronal damage or death (Marsman et al., 2014). Postnatal blockade of NMDAR causes impairment of fear-related emotional memory in rodents and sensory gating deficit in adolescence and adulthood, which may be potentially translated as a risk indicator or marker for schizophrenia onset (Latusz et al., 2017). Similarly, in support to the aforementioned statement, Mintz and Kopelowicz (2007) suggested that cognitive symptoms often precede psychosis and its treatment could be a better predictor of prognosis. Cognitive deficits on tests indexing verbal and visual knowledge or processing speed and working memory are also apparent in children who later develop schizophrenia (Reichenberg et al., 2010). In addition, a premorbid reduction in lower intelligence quotient (IQ) by ~0.5 standard deviation may also be a predictor of a later diagnosis of schizophrenia (Woodberry et al., 2008; Kendler et al., 2015). These cognitive deficits are noted to emerge early and, in most cases, stably persist without deterioration as the disorder progresses (Birnbaum and Weinberger, 2017). GABAergic inhibitory neurons have long been proposed as a promising therapeutic target for schizophrenia (extensively reviewed by Xu and Wong, 2018) though a great challenge remains. For example, Bojesen et al. (2020) have recently observed a reduced GABA level in the dorsal anterior cingulate cortex (dACC) in antipsychotic-naïve schizophrenia patients but failed to demonstrate its association with cognitive performance.

A reduced GABA level in patients with schizophrenia also associates with lower intelligence quotient (IQ) compared to healthy controls (Marsman et al., 2014), which suggests that the GABA deficit hypothesis principally applies to this group of patients (Hedman et al., 2013). GABAergic interneurons’ function and connectivity vary greatly from region to region in the brain, and their abnormalities also vary across various regions: GABAergic cells in layer III of the dorsolateral prefrontal cortex (DLPFC) and layer II of the anterior cingulate cortex (ACC) specifically show laminar alterations in schizophrenia patients (Benes, 2015). A bulk of evidence suggests that altered GABAergic transmission may be one of the root causes of schizophrenia. Here, the roles of GABAergic interneurons in schizophrenia pathology have been widely discussed and the existing mechanistic loops in understanding have also been highlighted.

## Gabaergic Interneurons and Schizophrenia

**FIGURE 1:**
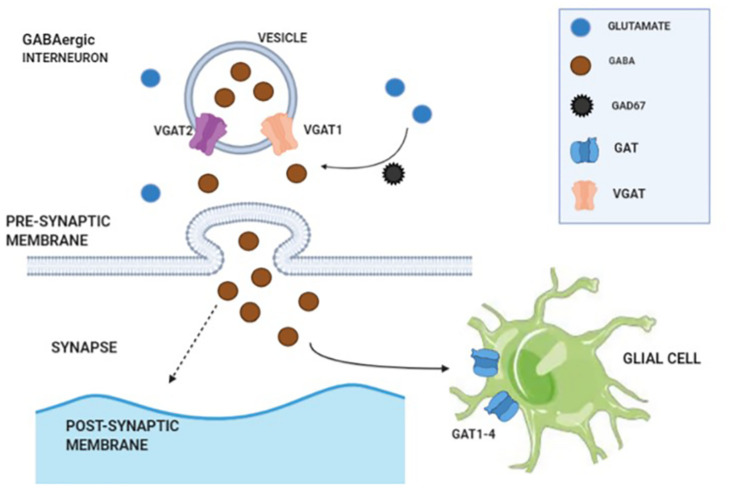
Illustration of γ-amino butyric acid (GABA) synthesis, transmission, and reuptake from synaptic cleft.

Cortical GABAergic interneuron is one of the key elements to coordinate cognitive processes and complex emotions via the release of γ-amino butyric acid (GABA). Glutamate serves as a precursor for GABA synthesis, and the reaction is mediated by glutamate decarboxylase enzymes (GAD65 and GAD67). GABA is stored in cytoplasmic vesicles via vesicular GABA transporters (VGAT1 and VGAT2). Upon release into synaptic cleft, the GABAergic signaling can be terminated in two ways: (1) GABA uptake by presynaptic terminals via plasma membrane GABA transporters (GAT1–4) and (2) GABA uptake by glial cells (via GAT1–4) (Fogaça and Duman, 2019; illustrated in Figure 1). Further release of GABA needs stimulation of GABAergic interneurons via an excitatory input. A reduced level of glutamate decarboxylase enzyme 67 (GAD67) in the post-mortem brain tissue of patients with schizophrenia is consistently reported in literature. Among the various brain regions, the dorsolateral PFC has been mostly found with a reduced expression of GAD67 messenger RNA (mRNA), a consistently replicated pathological disturbance in schizophrenia (Akbarian and Huang, 2006; Gonzalez-Burgos et al., 2010).

**FIGURE 2:**
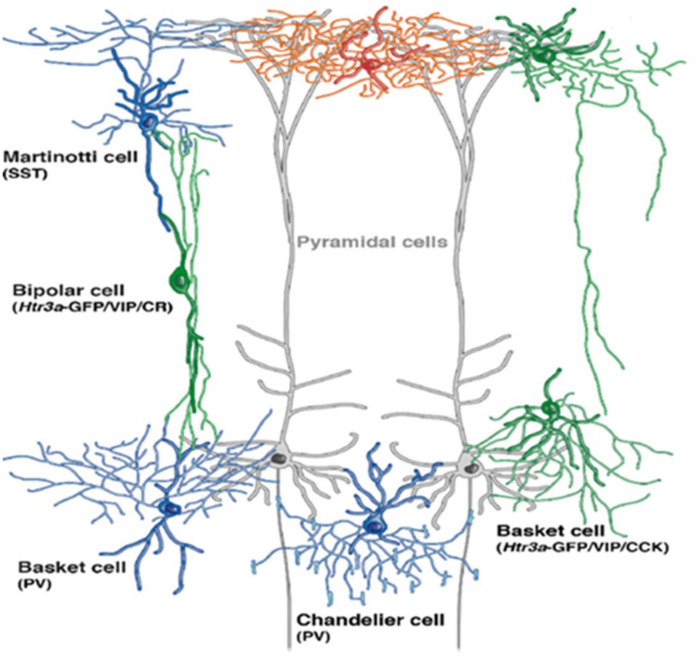
Depiction of GABAergic interneuron’s location within the cortex (adopted a cropped version from Niquille et al., 2018, under the terms of Creative Commons Attribution License). Gray extended cells are pyramidal cells spanning across cortical layers. Basket cells are aligned to deliver their transmissions to cell bodies of pyramidal neurons, whereas chandelier cells deliver inputs on axon initial segment (AIS) of principle neuron.

GABAergic interneurons are categorized into various subtypes based on the following: (1) morphology (chandelier cells or basket cells) (Figure 2); (2) electrophysiological properties (low-threshold spiking or fast-spiking); (3) synaptic connectivity (with distal dendrites, axon, or soma of postsynaptic neuron); and (4) on the basis of gene expression, typically the calcium-binding proteins (calretinin, calbindin, somatostatin, and parvalbumin) or neuropeptides: neuropeptide Y, vasoactive intestinal peptide (VIP), cholecystokinin, somatostatin, and 5-HT_3__A_ receptors expressing interneurons but infrequently contain more than one of these markers. To conclude, there are three distinct types of interneurons with non-overlapping markers [parvalbumin (PV), somatostatin (SST), and 5-HT_3__A_ receptors]. Interneurons are extended vertically across cortical layers (translaminar), horizontally (transcolumnar), or restricted locally to a single layer oriented horizontally, which has been extensively elaborated by Tremblay et al. (2016). Apart from maintaining normal cortical function, during postnatal development, GABAergic neurons play a crucial role in proper maturation of neural circuits (Nakazawa et al., 2012) as GABAergic neuronal circuits are immature at the time of birth (Chattopadhyaya, 2004). Maturation of GABAergic innervation patterns prolongs up to the post-adolescent period in primate’s prefrontal cortex (Cruz et al., 2009), and GABAergic system hypo-functioning commonly predisposes to schizophrenia (Lewis, 2014; Ferguson and Gao, 2018a). Among the post-mortem analyses of neurotransmitter dysfunction, GABAergic dysfunction is the only consistent finding in patients with schizophrenia (Birnbaum and Weinberger, 2017). Disrupting GABA signaling during early development alters cellular migration and cortical architecture in cell type-dependent ways in rodents (Manent et al., 2007; Cuzon et al., 2008; Thompson et al., 2009; Aronne et al., 2011; Wu et al., 2012; Haas et al., 2013). For example, GAD1 suppression during adolescence significantly decreased axonal branching in PV + cells in a cell-autonomous manner (Chattopadhyaya et al., 2007) and increased pyramidal cell activity (Lazarus et al., 2015).

It is understood that by early adulthood, cortical circuits are refined through the pruning of excitatory synapses, elaboration of inhibitory circuits, and remodeling of pyramidal dendrites. The discovery of effective therapeutic strategies is yet awaited to rescue postnatal maturation hindrances and over-pruning and to maintain a healthy GABAergic system to alleviate the risk of developing and also the developed schizophrenia.

### Parvalbumin Interneurons: A Strong Candidate for Schizophrenia Pathology

Parvalbumin (PV) interneurons are fast-spiking neurons containing a sufficient number of mitochondria to compensate for high demand of energy (Kann et al., 2014) and are classified into basket cells (providing perisomatic inhibition that is well-suited for feedback inhibition and output regulation) and chandelier cells that innervate axon initial segments (AIS) of pyramidal cells (Markram et al., 2004) as well as other interneurons (Lovett-Barron et al., 2012). It is suggested that PV basket cells and chandelier cells are electrically connected *via* gap junctions (Hestrin and Galarreta, 2005; Woodruff et al., 2011), which implicates that they may influence pyramidal cells in a synchronized way. Apart from PV basket cells, cholecystokinin (CCK) large basket cells also provide perisomatic inhibition to principal cells, though their properties are contrasting to PV FS basket cells (Klausberger et al., 2005; Glickfeld and Scanziani, 2006; Freund and Katona, 2007). Whereas, in the hippocampus, the CCK interneurons are also found targeting dendrites (Cope et al., 2002). However, there is a distinction in the connectome of PV and CCK interneurons. PV basket cells receive input from local pyramidal cells (PCs) to regulate spike timings and precision of cortical network oscillation. Whereas, CCK interneurons’ activities depend on subcortical inputs carrying information about mood and autonomic state of animals (Freund and Katona, 2007).

Each PV interneuron innervates hundreds of pyramidal neurons and regulates their output and synchrony (Williams et al., 1992; Cobb et al., 1995; Miles et al., 1996), energizes appropriate behavioral responses, and has largely diminished function in a schizophrenic brain (Ferguson and Gao, 2018a). Functional impairment of both types of PV (basket and chandelier) containing interneurons including the reduced expression of parvalbumin, GABA transporter (GAT)-1 mRNAs, and Gad1 (GAD67) all contribute to schizophrenia development (Lewis and González-Burgos, 2008). PV interneurons are extremely vulnerable to oxidative stress during the period of postnatal maturation when their fast-spiking properties are not yet fully established (Yang et al., 2014). In varieties of animal models of schizophrenia, oxidative stress has been found negatively correlated with the cell number of PV interneurons along with reduced neurite elaboration. Animal model studies showed that peri-adolescence administration of antioxidants can re-establish or guard the integrity of PV interneurons. This implicates that the antioxidant-based therapy in initial phase may be beneficial for healthy PV interneuron-associated networks and enhance cognitive processing or improve general performance of patients (Steullet et al., 2017). In the context of aforementioned study by Steullet et al. (2017), questions that have arisen and need to be addressed are as follows: Can peri-adolescence administration of antioxidants be a prophylactic choice? How can we identify the markers for oxidative stress that particularly serve as a signal for schizophrenia onset?

PV interneurons regulate and maintain excitatory and inhibitory balance in the cortex to control the targeted neuron’s activity in a homeostatic range. Spiking outputs from pyramidal neurons to targeted brain areas are regulated by PV interneurons (Fogaça and Duman, 2019). The thalamus is the major source of cortical excitatory inputs, which branch out and synapse on excitatory and inhibitory neurons of the cortex (Ferguson and Gao, 2018a). Electrical stimulation of thalamic neurons causes a direct excitatory response in cortical pyramidal neurons, but hyperpolarization occurs shortly due to the activation of neighboring PV basket cells (Agmon and Connors, 1991; Castro-Alamancos and Connors, 1997). This suggests that PV interneurons regulate the spike timings in neighboring pyramidal neurons across cortical regions (Wehr and Zador, 2003). It also highlights that PV interneurons may play a temporal and critical functional role in executive performance. Furthermore, an optogenetic approach also demonstrates the crucial role of PV interneurons in the generation and maintenance of gamma oscillation (Cardin et al., 2009; Sohal et al., 2009), which have been found significantly altered in schizophrenia (McNally and McCarley, 2016). Optical tagging unraveled that PV interneurons provide a more rapid and synchronous inhibition of excitatory neurons in the medial prefrontal cortex (mPFC) compared to a weak inhibition by SST interneurons (Kvitsiani et al., 2013). Similarly, the fast spiking of PV interneurons in the mPFC increases the firing rate during attentional processing (goal-directed behavior) (Kim et al., 2016). But, disrupting the excitatory and inhibitory balance by excessive activation (pharmacological) of PV interneurons in the mPFC considerably impairs cognitive flexibility, working memory, and social interaction (Ferguson and Gao, 2018b), whereas social interaction and working memory deficit can be compensated by increased pyramidal neuron excitability (Kamigaki and Dan, 2017). Chemogenetic inhibition of PV interneurons in the ACC alleviates observational fear (empathy in rodents) in mice but not the direct fear learning (Zhou et al., 2018), which mimics the feature of lack of empathy in schizophrenia patients. Hence, targeting PV interneuron function can be one of the effective therapeutic options.

### Somatostatin Interneuron’s Role in Schizophrenia Pathology

Somatostatin (SST)-expressing Martinotti cells are low-threshold spiking interneurons that project expansively to distal dendrites of pyramidal cells to control incoming cortical signals (Kawaguchi and Kubota, 1997). Increased dendritic inhibition by SST over perisomatic inhibition by basket cell (parvalbumin-positive) inhibition could lead to more robust filtering of task-irrelevant information (Anderson et al., 2020). Vasoactive intestinal peptide (VIP, 5-HT_3__a_ receptor expressing) interneurons inhibit both the PV and SST interneurons and are implicated as an important disinhibitory player in cortical circuits (Pi et al., 2013). SST cells control the spiking inputs to pyramidal neurons (Fogaça and Duman, 2019). On the other hand, rhythmic dendritic inhibition of pyramidal neurons by SST interneurons represents an alternative mechanism for the generation or maintenance of gamma rhythms (Veit et al., 2017). Furthermore, SST interneurons are specifically activated by threat-associated cues, and its activity is required for cued memory expression, not for context fear. Additionally, SST interneurons have inhibitory transmission onto PV interneurons, which in turn disinhibits principal neurons to elicit fear expression. SST interneurons preferentially mediate the encoding of cue-associated signals. Presumably, SST interneurons are strongly activated by the basolateral amygdala (BLA) afferents following conditioning and that circuit plasticity may favor their recruitment over PV interneurons (Cummings and Clem, 2020). It should be noted that although research regarding schizophrenia focus on PV interneurons as a reason for pathophysiology, the functions of SST interneurons are also widely affected in the prefrontal cortex in the patients. A recent study has demonstrated a reduced SST interneurons’ density in schizophrenia patients and profound laminar deficit with an increase of death receptor (apoptotic activity). This means that SST interneurons are vulnerable to the elevated death receptor, which can result in putative neuronal loss (specifically in layer II of the cortex). The possible greater pathology in SST mRNA-containing neurons as compared with PV-containing neurons in people with schizophrenia may be a key finding for consideration when developing relevant animal models for schizophrenia (Joshi et al., 2015). Glutamatergic hyperactivity, oxidative stress, and apoptosis have been associated with cortical neuronal loss. However, how to manage or cease the progressive apoptotic activity in patients yet remains for further investigation.

A reduced expression of SST has also been reported within the dorsolateral prefrontal cortex in patients with depression (Sibille et al., 2011), which is also commonly affected in schizophrenic brain. Shared molecular substrates make it more challenging to distinct the molecular pathology for psychiatric disorders. Moreover, a recent publication reported that a decreased SST (but not PV) mRNA expression in the ventral hippocampus is enough to cause reduced social interaction (negative symptom of schizophrenia), while knockdown of both PV and SST in the mPFC significantly decreased interaction time during a social interaction test. Furthermore, PV and SST interneurons in the mPFC contribute to cognitive flexibility (Perez et al., 2019), which implies the region-specific function or division of labor and function-specific manner of GABAergic interneurons. In contrast, another recent study reported that stem cell-derived PV interneuron (not SST) transplantation in the ventral hippocampus can reverse the deficit in a MAM schizophrenia rodent model (Donegan et al., 2017). If the concept is extrapolated further, yet it is needed to unravel the differential role of GABAergic interneurons, how do they produce different effects with same neurotransmitter? Does their oscillation vary or show region-specific differences?

Medium spiny neurons are GABAergic interneurons abundantly presented in the striatum and basal ganglia. Recently, the association of medium spiny neurons with schizophrenia has been evidenced, which is independent from cortical interneurons and pyramidal neurons. The genetic risk associated with medium spiny neurons does not overlap with that of glutamatergic pyramidal cells and cortical interneurons, indicating that different cell types have biologically distinct roles in schizophrenia (Skene et al., 2018). Further research in this area will help to identify specific cell types responsible for schizophrenia and target-based therapy.

Notably, schizophrenia has shared molecular neuropathology with bipolar and autism spectrum disorders, and bipolar presents a similar synaptic dysfunction with schizophrenia (Gandal et al., 2018), which increases the chances of diagnostic misclassification. It needs to be warranted in the future to determine whether neuronal firing rate differences exist across psychiatric diseases as it could serve as a potential marker for distinct disorders.

## Neural Oscillations and Schizophrenia

Orchestrated neural oscillations are fundamental to establish precise temporal relationships between neuronal responses important for memory, perception, and consciousness. In patients with schizophrenia, the beta- and gamma-band activity is dis-synchronized, suggesting a crucial role for dysfunctional oscillations in the generation of the cognitive deficits and other relevant symptoms. Dysfunctional oscillations may arise owing to anomalies in the brain’s rhythm-generating networks of GABAergic interneurons and in cortico-cortical connections (Uhlhaas and Singer, 2010). Neural oscillations in the low (theta and alpha) and high (beta and gamma) frequency ranges establish precise temporal correlations between distributed neuronal responses. Oscillations in the beta and gamma range establish synchronization with great precision in local cortical networks (Womelsdorf et al., 2007), whereas lower frequencies preferentially establish synchronization over longer distances. Synchronized oscillations have been shown to establish the precision in spike timing that is crucial for use-dependent synaptic plasticity (Wespatat et al., 2004).

Reduced synaptic connectivity is sufficient to produce a large deficit in gamma oscillations, and gamma (γ) deficit in schizophrenia patients could be a marker of reduced synaptic connectivity underlying cortical network. Though gamma deficit would not be indicative of the type of the underlying synaptic connectivity abnormality, it has been suggested that gamma oscillations may be a more sensitive indicator of circuit integrity than MRI measurement, as a 10% decrease in synaptic connectivity is enough to produce a substantial gamma deficit (Spencer, 2009). The blood oxygen level-dependent (BOLD) response primarily reflects the metabolic demand of the synaptic input to a cortical area instead of its spiking output (Viswanathan and Freeman, 2007). Reduced synaptic connectivity in targeted cortical area usually indicates fewer synaptic input, reduced metabolism, and decreased BOLD response (Spencer, 2009), which indicates the synaptic input could be harnessed to increase the BOLD response. Moreover, it is evidenced that the BOLD response is positively correlated with gamma oscillations (Niessing et al., 2005; Scheering et al., 2016), which supports that microcircuit abnormalities can precipitate altered gamma oscillations that may produce similar changes in BOLD response (Spencer, 2009).

Source analyses revealed that in schizophrenia patients, the gamma-band oscillations triggered by transcranial magnetic stimulation (TMS) did not propagate beyond the area of stimulation, whereas in controls, it can be found in multiple motor and sensorimotor regions. Patients with schizophrenia also demonstrate reduced amplitude and less synchronization of self-generated, rhythmic activity in several cortical regions. The absence of theta coherence between left frontal and temporal electrodes suggests a failure in the preparation of temporal areas for speech production, which could lead to the misattribution of self-generated speech to an external source (Uhlhaas and Singer, 2010). Modification of synaptic contacts depends on the precise temporal coordination of neural activity. Aberrant neural oscillations during early critical periods may lead to disorganized temporal coordination of neural activity and result in the pathological modification of cortical circuits (Uhlhaas and Singer, 2010).

PV interneurons display a fast-spiking pattern of firing, which is not only crucial for temporal control of cortical inhibition but also generates synchronous membrane potential gamma band oscillations (30–80 Hz) (Nakazawa et al., 2012) relating to information and cognitive processing across species (Buzsáki and Draguhn, 2004). Optogenetic approaches revealed that light-driven activation of PV-expressing, fast-spiking interneurons selectively amplifies gamma oscillations (Cardin et al., 2009; Sohal et al., 2009). Whereas, gamma power is diminished when excitatory drive onto PV neurons is selectively reduced (Fuchs et al., 2007). However, inhibition of excitatory neurons by PV-containing basket cells synapsing on perisomatic regions of excitatory neurons is essential for the synchronization of neural activity (Bartos et al., 2007; Mann and Paulsen, 2007). Alteration in PV interneuron-mediated inhibition is implicated in defective gamma band synchrony in schizophrenia (Gonzalez-Burgos and Lewis, 2012). The finding that gamma band oscillations are dependent upon GABAergic interneurons is compatible with a large body of evidence suggesting alterations in GABAergic neurotransmission in schizophrenia, including a reduction in the messenger RNA (mRNA) GAD67 and functional deficits of PV interneurons (Lewis et al., 2005).

The EEG results also demonstrate that synchronized oscillatory activity, particularly in the gamma range (30–80 Hz), is abnormal in schizophrenia patients (Ferrarelli et al., 2008). These oscillations, either low or high frequency, are reduced even in resting state, which could be explained by functional dysconnectivity. Reduced beta oscillation preceding disrupted gamma oscillations may serve as a clue for the onset of schizophrenia. Transcranial alternating current stimulation (tACS) not only enhanced and modulated the reduced alpha oscillation but also improved functional connectivity and reduced auditory hallucination in schizophrenia patients (Ahn et al., 2018).

Myelination of fast-spiking interneurons (FSIs) has crucial roles in regulating the function of FSI. Hence, the defect in FSI myelination likely alters local cortical circuit oscillations *in vivo* that, as a result, contributes to cognitive deficits (Benamer et al., 2020) in schizophrenia (Maas et al., 2020). Therefore, myelination of PV interneurons is vital for the normal functioning of mature cortical inhibitory circuit (Benamer et al., 2020). Moreover, it should be considered that multiple sclerosis also shares overlapping genetics with schizophrenia (Arneth, 2017), which is however largely ignored. Poor myelination or lack of myelin sheath disturbs the information flow and the production of neuronal oscillations. Impaired myelination of PV interneurons could directly contribute to schizophrenia through the following mechanisms: energy restrictions during highly demanding cognitive tasks, aberrant axonal branching, and impaired action potential fidelity, which consequence into disrupted inhibitory network function. Such changes to PV interneurons would likely result in abnormalities of local gamma synchronization, which most likely influences the integrity of long-range thalamo-cortical and cortico-striatal circuits and striatal dopamine signaling, eventually precipitating the symptoms of schizophrenia (Stedehouder and Kushner, 2017). The late adolescent age of onset for schizophrenia closely overlaps with the maturation of prefrontal cortex myelination (Do et al., 2015), which implicates that poor myelination is one of the causes of schizophrenia pathology. Independent of PV interneuron alterations, myelination abnormalities have also been extensively implicated in schizophrenia through both *in vivo* brain imaging and post-mortem examination (Stedehouder and Kushner, 2017). However, cell type-specific myelination studies in the post-mortem human brain are lacking, which needs attention for future post-mortem investigation. Though multiple sclerosis can be induced in mice, it is yet to know which brain areas or cell types should be targeted in order to produce a schizophrenia model with phenotypes of multiple sclerosis.

## Imaging Approaches

Imaging technique is a non-invasive approach to investigate different brain regions providing an activity-based picture of the brain. Various dysfunctional or hyperactive/hypoactive brain regions serve as the cause of disease, which can be differentiated easily via imaging. Nelson et al. (2015) has reported a hypoactivity of the anterior cingulate cortex (ACC) during emotion processing in schizophrenia patients. Functional imaging approaches suggested that patients with schizophrenia exhibit hypofunction in the PFC (Van Snellenberg et al., 2016), and reduced volume of the frontal cortex is also confirmed in schizophrenic post-mortem tissue (Selemon et al., 2002), and enlargement of the lateral and third ventricles and smaller cortical and gray matter volumes are also commonly found in patients with schizophrenia (Nelson et al., 1998; Wright et al., 2000).

Proton MR scanner (^1^H-MRS) is a non-invasive approach to identify and quantify certain biochemical compounds in brain tissues (Zhang et al., 2013). As opposed to post-mortem studies, Kegeles et al. (2012) have reported an elevated GABA level in the mPFC in unmedicated patients via proton MR scanner (^1^H-MRS). It is mostly likely that the elevated GABA level in the mPFC could be the overcompensation of a subclass of GABAergic neurons rather than PV interneurons (Kegeles et al., 2012). The deficits in GABAergic (fast-spiking interneurons) transmission upon pyramidal neurons lead to glutamate elevation (Lewis and Moghaddam, 2006; Lisman et al., 2008), which in turn stimulates the rest of the subclasses of GABAergic interneurons to compensate the reduced GABA synthesis of PV neurons (Kegeles et al., 2012). Moreover, spatial distributions of interneuron subtypes could underlie regional signaling differences across the cortical sheet, as BOLD functional magnetic resonance imaging (Anderson et al., 2020) has suggested a heterogeneous distribution and region-specific function of interneurons. A recent study using ^1^H-MRS also showed reduced GABA level in the ACC in schizophrenia or psychosis. However, the main effects of GABA on spatial working memory, attention, and premorbid intelligence were unfortunately found insignificant (Bojesen et al., 2020). Moreover, a meta-analysis using ^1^H-MRS has revealed inconsistent alteration of GABA level in schizophrenia (Egerton et al., 2017). This has made it more challenging to precisely associate GABA reduction with schizophrenia pathology, as the actual GABA level varies across human brain regions. Further research can open a new window to unravel the region-based function and cytoarchitecture differences of GABAergic interneurons across brain regions and even the variations from patient to patient, which will ultimately provide a road map toward personalized medicine.

## Animal Models

Animal models mimicking human psychiatric disorders have an obvious and utmost importance to understand the neurobiological mechanism of these disorders and are also useful tools for preclinical trials. They serve as a rapid platform to monitor disease progression. Moreover, animal models offer opportunity for invasive monitoring of molecular and structural changes, which serves as bases for disease and novel therapeutics. Schizophrenia animal models can be produced via four approaches: lesion, drug induced, developmental, and genetic manipulation. For extensive details, one may see a review by Jones et al. (2011). Nevertheless, there is a scientific challenge to evaluate and quantify some of the core symptoms of psychiatric disorders (like verbal learning, memory, and thoughts), which are exclusively human traits (Powell and Miyakawa, 2006) or maybe animals do have thoughts and we are not aware of and not able to assess. Acute and chronic administration of ketamine in mice cannot mimic schizophrenia completely, but a chronic ketamine model can display schizophrenia-like phenotypes (Behrens et al., 2007; Phoumthipphavong et al., 2016). Chronic administration (7–14 days) of ketamine affects NMDA receptor-mediated neurotransmission and also recapitulates some schizophrenia pathophysiology such as alterations of PV interneurons (Behrens et al., 2007), gamma oscillations (Sullivan et al., 2015), cognition (Featherstone et al., 2012), and dendritic spines (Phoumthipphavong et al., 2016).

The schizophrenia Df(16)A+/− model with 22q11.2 microdeletions (a highly penetrant genetic variant for schizophrenia) and the chronic ketamine administration model both produced deficits in stimulus-elicited gamma power analogous to those reported in sensory cortices of human SZ patients (Hamm et al., 2017). Interestingly, Belforte et al. (2010) had created a conditional knockout mouse strain in which the NR1 subunit was selectively ablated in approximately half of cortical and hippocampal GABAergic neurons, a majority of which contain parvalbumin. The mutants exhibited reduced GAD67 and PV levels in the cortical GABAergic neurons with NR1 deletion and presented deficits in spatial working memory and PPI, which could be ameliorated by the antipsychotic risperidone. In contrast, post-adolescent deletion of NR1 subunit in the same interneuron population did not result in schizophrenia-like abnormalities, demonstrating a fundamental role of NMDARs during the early postnatal stages with regard to the development of schizophrenia-like phenotypes in later adulthood. In summary, this model not only produces many aspects of schizophrenia but also mirrors three additional characteristics of human schizophrenia: stress-dependent precipitation of symptoms, a latency period before the development of symptoms, and a critical period for disease acquisition. It also exhibits non-behavioral features (such as decrease in GAD67 and PV expressions) compatible with schizophrenia, increasing the face validity of the model. These results suggest that in schizophrenia, NMDAR hypofunction in the cortical GABAergic interneurons is one of the major “shared” pathophysiological pathways originating from malnutrition, infection, obstetric complications during development, and a variety of other possible etiological factors (Belforte et al., 2010; Nakazawa et al., 2012). However, it is still unclear which brain region is the ultimate target and involved in the pathology or which areas share the burden or play discretely for the disease development. Secondarily, cognitive impairment often precedes the onset of schizophrenia, and in the context of relevant studies (Mintz and Kopelowicz, 2007; Belforte et al., 2010; Latusz et al., 2017), would it be a possible therapeutic option to boost NMDAR activity in order to alleviate the clinical symptoms? Does memory impairment itself serve as a precursor for the progression of schizophrenia? Will the oscillations of memory-impaired subjects provide a clue for the onset of schizophrenia? Further research is warranted to replicate these experiments to provide clearer continuum.

To date, a couple of rodent models have been created to mimic altered GABA levels in human patients. For example, a global GAD1 knockout rat model was recently developed using CRISPR/Cas9 genome editing technology. The rats successfully re-capitulated cognitive impairment and behavioral alterations relevant to negative and positive symptoms of schizophrenia. However, the mutants cannot mimic the physical GAD67 level in patients due to the complete depletion of GAD67 (Fujihara et al., 2020). As opposed, a mouse model with GAD1 genetically ablated within ~50% cortical and hippocampal interneurons resulted in a subset of negative symptoms with reduced willingness to expend costly effort, as noticed in patients with schizophrenia (Kolata et al., 2018). Mice heterozygous for GAD67 deficiency primarily in PV neurons also exhibited schizophrenia-like abnormalities such as deficits in pre-pulse inhibition, social memory, and reduced inhibitory synaptic transmission (Fujihara et al., 2015). Additionally, knockdown of GAD1 in PV interneurons also led to noticeable sensory motor gating deficits and reduced fear extinction in mice (Brown et al., 2015). Currently, rodent models being used for the schizophrenia research are of varied degrees of face validities. Structural differences between human and rodent brain hugely impede the tracing of the precise causes or the replication of the full set of core phenotypes. The PFC controls cognitive and executive functions that are disrupted in schizophrenia, but the major structural and functional differences exist between the human PFC and the medial frontal cortex of rodents. From this point of view, the common marmoset is emerging as a promising model to integrate both construct and face validity through genetic, behavioral, functional circuitry, and histological analyses. As a primate, the marmoset PFC also undergoes peri-adolescent pruning of dendritic spines and thus closely resembles the human PFC in anatomy and function (Kanyuch and Anderson, 2017). Based on these advantages, the utility of marmoset-based disease model may shed more insights for fundamental neuropathologies and provide a much clearer picture for the development of specific therapeutics.

## Stem Cell-Based Therapeutic Approaches

Disorder-specific human-induced pluripotent stem cells (hiPSCs), which bear the genetic makeup of donor patient and can differentiate into specific cell types of interest, are one of the tools to study or even to compensate the damages leading to schizophrenia (Grunwald et al., 2019). A comparison between neuronal cells derived from healthy and diseased individuals has provided important insights into the molecular and cellular basis of schizophrenia (Ahmad et al., 2018). A recent study has shown that neurons derived from somatic cells (skin cells) of schizophrenia patients displayed reduced neurite growth and down-regulated synaptic scaffolding protein PSD95. However, it has been suggested that neurite outgrowth may not be the best parameter to define the beneficial effects of antipsychotic drugs on iPSCs derived from schizophrenia patients, as neurite under-growth has also been observed in autism spectrum disorder. An assessment of calcium responses in cultured cells and transcriptomic profile can however serve as tools to discriminate between schizophrenia and autism. Proteomic analysis revealed that the expression of vacuolar protein sorting-associated protein 35, involved in retrograde transport to the Golgi apparatus, is downregulated by ~75% in schizophrenia (Grunwald et al., 2019). The reduction in vacuolar protein sorting-associated protein 35 reflects the disturbed synaptic vesicles transportation within hiPS cells. A similar pattern of disruption has been confirmed in post-mortem cerebellar tissue of schizophrenia patients (Mudge et al., 2008). Decreased neuronal connectivity is also detected in schizophrenia patient-derived (SCZD) hiPSC neurons, which can be rescued by loxapine, though the reduced connectivity cannot fully explain the synaptic dysfunctioning. Notably, currents across cell membrane for sodium, potassium and calcium transients and excitatory and inhibitory currents were not affected in SCZD hiPSC neurons. Moreover, neuregulin 1 (NRG1) expression is found increased in SCZ hiPSC neurons only, not in neural progenitor cells, fibroblast cells, or hiPSCs, which indicate the cell-specific gene expression alteration relevant to disorder (Brennand et al., 2011). NRG1 is one of the 108 schizophrenia-associated identified genes (Ripke et al., 2014) and is significantly associated with endophenotypes of schizophrenia via regulating myelination (Chen et al., 2006), neuronal migration (Ghashghaei et al., 2006), and function of neurotransmitter receptors (Liu et al., 2001; Hahn et al., 2006). Post-mortem studies have revealed reduced brain volume, cell size, spine density, and abnormal neural distribution in the prefrontal cortex and hippocampus of SCZD brain tissue (Brennand et al., 2011). Reduced dendritic arborization has been observed in post-mortem SCZD brains (Selemon and Goldman-Rakic, 1999) and in animal models (Jaaro-Peled et al., 2010). Whereas, PV and SST interneuron transplantation into the ventral hippocampus and medial prefrontal cortex abolishes the deficits presented by MAM model (Donegan et al., 2017; Perez et al., 2019). PV and SST interneuron transplantation is an effective therapeutic option that not only compensates the deficit but also helps to re-establish neural circuitry or arborization in the instinct manner. Transplantation of brain tissue has some limitations: (1) the life span of transplanted tissue is uncertain or not long enough, (2) the source of stem cells (mainly from human fetal tissue) is an obstacle, and (3) it is hard to manage the migration of transplanted stem cells into the desired areas in adulthood. Importantly, it remains debatable whether the subventricular niche still retains robust neurogenic capacity in adult human brain as the current research seem controversial (Sanai et al., 2011; Wang et al., 2011).

## Conclusion

To conclude, the reduced neural connectivity, brain volume, and spine density have been reported as commonly agreed endophenotypes of schizophrenia, but the reduction in synaptic function has not yet been studied in detail. In addition, the perturbed information flow among different brain regions is common for many psychiatric disorders, but schizophrenia can be assessed more precisely via transcriptome analysis. Moreover, demyelination of PV interneurons, which in turn reduces gamma oscillations, also promotes the onset of schizophrenia. It remains unclear how to regulate the postnatal maturation and migration of inhibitory neurons, over-pruning of excitatory synapses, pyramidal dendritic remodeling, and deciphering the underpinning mechanisms, which may alleviate the process of or even the onset of schizophrenia. Importantly, studies need to be warranted in the future to target cell type-specific remyelination, synaptogenesis and neurite outgrowth. The literature regarding GABA alterations is heterogeneous in terms of GABA level and also the affected brain areas. Therefore, so far, a commonly agreed mechanism is lacking. Apart from genetic predisposition, perhaps, each type of environmental insult is hitting different areas in the brain. It is speculated that each cell type of GABAergic system probably responds differently to environmental stress. It is also assumed that possibly each type of environmental stress (nature of stress) has different effects on different cell types. Designing animal studies regarding exposure to various environmental insults with various intensities on different times would help to answer the speculations. Moreover, there is a scope for developing schizophrenia animal model with cell-specific demyelination to probe that either cell-specific sensitivity occurs. Furthermore, *in vivo* multicellular calcium imaging, for cells integrated in circuit, in an animal model would help to identify the interaction between GABAergic, glutamatergic, and dopaminergic systems to delineate the landscape of interconnecting mechanistic pathways.

## Author Contributions

BL and X-PW conceived the idea. All authors contributed, read and approved the final manuscript.

## Conflict of Interest

The authors declare that the research was conducted in the absence of any commercial or financial relationships that could be construed as a potential conflict of interest.
