# Corrigendum: GABAergic System Dysfunction and Challenges in Schizophrenia Research

**DOI:** 10.3389/fcell.2021.742519

**Published:** 2021-08-13

**Authors:** Muhammad Jahangir, Jian-Song Zhou, Bing Lang, Xiao-Ping Wang

**Affiliations:** ^1^Department of Psychiatry, National Clinical Research Center for Mental Disorders, The Second Xiangya Hospital of Central South University, Changsha, China; ^2^School of Medicine, Medical Sciences and Nutrition, Institute of Medical Sciences, University of Aberdeen, Aberdeen, United Kingdom

**Keywords:** schizophrenia, GABAergic system, oscillations, stem cells, animal models

In the original article, we neglected to include the funder “the Key Research and Development Program from Hunan Province (2018DK2011)” and “the National Natural Science Foundation of China (82071507)” to Bing Lang.

The authors apologize for this error and state that this does not change the scientific conclusions of the article in any way. The original article has been updated.

## Publisher's Note

All claims expressed in this article are solely those of the authors and do not necessarily represent those of their affiliated organizations, or those of the publisher, the editors and the reviewers. Any product that may be evaluated in this article, or claim that may be made by its manufacturer, is not guaranteed or endorsed by the publisher.

